# Prevalence and Evolution of Core Photosystem II Genes in Marine Cyanobacterial Viruses and Their Hosts

**DOI:** 10.1371/journal.pbio.0040234

**Published:** 2006-07-04

**Authors:** Matthew B Sullivan, Debbie Lindell, Jessica A Lee, Luke R Thompson, Joseph P Bielawski, Sallie W Chisholm

**Affiliations:** **1**Department of Civil and Environmental Engineering, Massachusetts Institute of Technology, Cambridge, Massachusetts, United States of America; **2**Department of Biology, Massachusetts Institute of Technology, Cambridge, Massachusetts, United States of America; **3**Department of Biology, Dalhousie University, Halifax, Nova Scotia, Canada; **4**Department of Mathematics and Statistics, Dalhousie University, Halifax, Nova Scotia, Canada; University of ArizonaUnited States of America

## Abstract

Cyanophages (cyanobacterial viruses) are important agents of horizontal gene transfer among marine cyanobacteria, the numerically dominant photosynthetic organisms in the oceans. Some cyanophage genomes carry and express host-like photosynthesis genes, presumably to augment the host photosynthetic machinery during infection. To study the prevalence and evolutionary dynamics of this phenomenon, 33 cultured cyanophages of known family and host range and viral DNA from field samples were screened for the presence of two core photosystem reaction center genes, *psbA* and *psbD*. Combining this expanded dataset with published data for nine other cyanophages, we found that 88% of the phage genomes contain *psbA,* and 50% contain both *psbA* and *psbD*. The *psbA* gene was found in all myoviruses and *Prochlorococcus* podoviruses, but could not be amplified from *Prochlorococcus* siphoviruses or *Synechococcus* podoviruses. Nearly all of the phages that encoded both *psbA* and *psbD* had broad host ranges. We speculate that the presence or absence of *psbA* in a phage genome may be determined by the length of the latent period of infection. Whether it also carries *psbD* may reflect constraints on coupling of viral- and host-encoded PsbA–PsbD in the photosynthetic reaction center across divergent hosts. Phylogenetic clustering patterns of these genes from cultured phages suggest that whole genes have been transferred from host to phage in a discrete number of events over the course of evolution (four for *psbA,* and two for *psbD*), followed by horizontal and vertical transfer between cyanophages. Clustering patterns of *psbA* and *psbD* from *Synechococcus* cells were inconsistent with other molecular phylogenetic markers, suggesting genetic exchanges involving *Synechococcus* lineages. Signatures of intragenic recombination, detected within the cyanophage gene pool as well as between hosts and phages in both directions, support this hypothesis. The analysis of cyanophage *psbA* and *psbD* genes from field populations revealed significant sequence diversity, much of which is represented in our cultured isolates. Collectively, these findings show that photosynthesis genes are common in cyanophages and that significant genetic exchanges occur from host to phage, phage to host, and within the phage gene pool. This generates genetic diversity among the phage, which serves as a reservoir for their hosts, and in turn influences photosystem evolution.

## Abstract 2

Analysis of 33 cultured cyanophages of known family and host range, as well as viral DNA from field samples, reveals the prevalence of photosynthesis genes in cyanophages and demonstrates significant genetic exchanges between host and phage.

## Introduction

The marine cyanobacteria *Prochlorococcus* and *Synechococcus* are the smallest and most numerous photosynthetic cells in the oceans [ 1, 2]. The abundances of cyanophages (cyanobacterial viruses) that infect these marine cyanobacteria vary over spatial [ 3– 6] and temporal scales [ 4, 7]—patterns shaped by the dynamics of their host cells [ 4, 8]. Cyanophages are double-stranded DNA viruses belonging to three morphologically defined families: Podoviridae, Myoviridae, and Siphoviridae [ 3– 5, 9, 10]. Among the cyanophages, podoviruses and siphoviruses tend to be very host-specific, whereas myoviruses generally have a broader host range, even across genera [ 5], and thus are potential vectors for horizontal gene transfer via transduction.

The movement of genes between organisms is an important mechanism in evolution. As agents of gene transfer, phages play a role in host evolution by supplying the host with new genetic material [ 11– 15] and by displacing “host” genes with viral-encoded homologues [ 16– 18]. Phage evolution is in turn influenced by the acquisition of DNA from their hosts [ 13, 19– 22] and by the swapping of genes within the phage gene pool [ 23, 24]. Recent evidence suggests that gene flow within the global phage gene pool extends across ecosystems [ 25– 27].

Cyanophage genomes bearing key photosynthesis genes *psbA* and *psbD* provide a notable example of the co-option of “host” genes for phage purposes [ 13, 22, 28– 30]. The *psbA* and *psbD* genes encode the two photosystem II core reaction center proteins, D1 and D2 (denoted here as PsbA and PsbD, respectively), found in all oxygenic photosynthetic organisms. It has recently been shown that the phage-encoded *psbA* gene is expressed during infection [ 31, 32]. Because maximal cyanophage production is dependent on photosynthesis [ 31, 33], and the host PsbA protein turns over rapidly [ 34] and declines during infection [ 31], expression of these phage-encoded genes likely enhances photosynthesis during infection, thus increasing cyanophage fitness.

If photosynthesis genes indeed provide a fitness advantage to cyanophages, one might expect them to be widespread among cyanophage genomes. Through whole or partial genome sequencing, *psbA* has been documented in three *Prochlorococcus* cyanophages (one podovirus and two myoviruses) and five *Synechococcus* myoviruses, whereas *psbD* was found in only some of these phages [ 13, 29, 35]. Neither of these genes is found in the *Synechococcus* P60 podovirus genome [ 36]. A survey of *Synechococcus* myovirus isolates revealed that at least 37 of them contained *psbA* [ 29], and this gene has also been found in cyanophage genome fragments in seawater samples [ 37]. Thus, the presence of *psbA* is a common, but not universal, feature in the cyanophages examined to date, most of which have been *Synechococcus* cyanophages.

Using limited genomic sequence data from one *Synechococcus* and three *Prochlorococcus* cyanophages, we suggested that both *psbA* and *psbD* were transferred as whole genes from host to phage multiple times, but not from phage to host [ 13]. Subsequently, Zeidner et al. [ 37] analyzed *psbA* data predominantly from field sequences and suggested that genetic exchanges of segments of the gene (intragenic recombination) may have occurred among host and phage copies in both directions [ 37]. However, this novel and controversial hypothesis requires further investigation with sequences of known organismal origin and using methodology capable of identifying the recombination partners and the directionality of such potential exchanges.

To better describe and understand the phenomenon of photosynthesis genes in cyanophage, we looked for the *psbA* and *psbD* genes in 33 cultured cyanophage isolates that infect *Synechococcus* or *Prochlorococcus* (or both) and analyzed the sequences of these genes in the context of known host ranges of the phage. This dataset allowed us to address the following questions: (1) How prevalent are both *psbA* and *psbD* in cyanophages that infect *Synechococcus* and/or *Prochlorococcus*? and (2) To what extent have photosynthesis genes, or segments thereof, been moved between and among hosts and phages?

## Results/Discussion

### Prevalence of the *psbA* and *psbD* Genes in Cyanophages

The *psbA* gene was amplified from 28 out of the 33 cyanophage isolates examined ( Table 1). Combining these findings with published results ( Table 1), we find that the *psbA* gene is present in 88% of cyanophage isolates examined, including all myoviruses ( *n* = 32) and all five *Prochlorococcus* podoviruses included in this study. However, this gene was not detected in *Prochlorococcus* siphoviruses ( *n* = 2) and *Synechococcus* podoviruses ( *n* = 3), suggesting that there are some combinations of phage family and host genus that do not lead to incorporation of the *psbA* gene into the phage genome. Six additional phages yielded ambiguous results and were excluded from these analyses (see Materials and Methods for details).

**Table 1:**
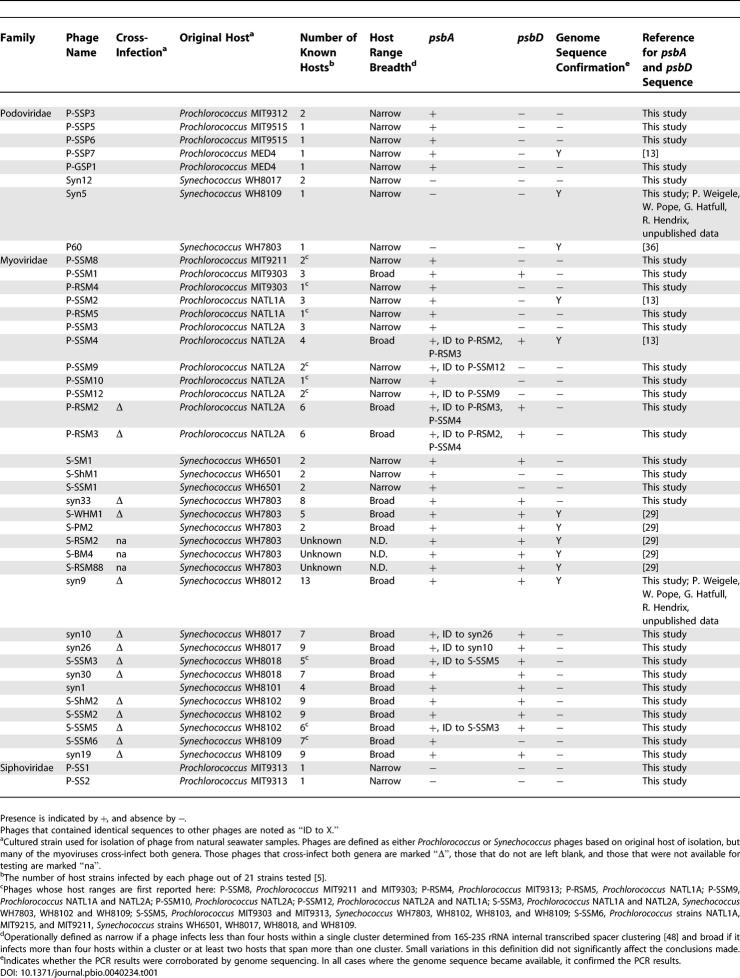
Presence or Absence of *psbA* and *psbD* among *Prochlorococcus* and *Synechococcus* Cyanophages

When present, the *psbA* gene is likely to be functional, as there is evidence for the conservation of amino acid sequences through purifying selection [ 13, 37], and the gene is expressed during infection [ 31, 32], implying that this gene confers a fitness advantage to the phages that carry it [ 13, 22, 29, 31]. Sustained photosynthesis is necessary for maximal phage production [ 31, 33, 38], and the long latent period of many freshwater and marine cyanophages (8 h or more; [ 9, 31, 33, 38]) presumably results in energy- and/or carbon-limitation for phage replication. Thus, cyanophage-encoded *psbA* likely serves to boost the photosynthetic performance of the host during infection, thereby increasing phage production. It is perhaps not coincidental that one of the phages that lacks *psbA*, *Synechococcus* podovirus P60 ( Table 1), has a latent period of only 1 h (K. Wang and F. Chen, personal communication), which may be too short for *psbA* expression to be beneficial. Latent period information for marine cyanophages, however, is sparse. It is not known for the *Prochlorococcus* siphoviruses that lack *psbA*, and it has only been shown to be >8 h for a single phage strain from each of the *Synechococcus* myoviruses [ 39] and *Prochlorococcus* podoviruses [ 31]. Further, theory [ 40– 43] and experiments [ 44] suggest that latent period length may be a transient property that rapidly evolves in response to changes in host cell densities. Thus, further exploration of this hypothesis requires analysis of the latent period of many more phage isolates under variable host cell concentrations.

The *psbD* gene was amplified from 15 out of the 33 cyanophage isolates examined ( Table 1). Again, combining our data with published findings, we observe that *psbD* is found only in isolates that contain *psbA* and only in myoviruses, but not in all *psbA*-containing myoviruses. Only four of 12 *Prochlorococcus* myoviruses (as defined by original host strain of isolation; Table 1) contained *psbD,* whereas this was the case for 17 of 20 *Synechococcus* myoviruses. Although it is possible that differences in the photosystem II reaction center between *Prochlorococcus* and *Synechococcus* exist (such as differences in the rate of PsbD degradation) and could explain the biased distribution of the *psbD* gene among the myoviruses, there is no evidence that this is the case. The breadth of phage host ranges (as operationally defined in Table 1), however, appears to be a reasonably good predictor of whether a phage will contain *psbD*: 17 of 18 broad-host-range phages encode it *,* whereas only one out of 21 narrow-host-range phages do so ( Table 1). Perhaps broad-host-range phages have co-opted both *psbA* and *psbD* to better ensure the formation of a functional PsbA–PsbD protein complex in the host during infection.

### Origins and Evolutionary History of *psbA* and *psbD* in Cyanophages

**Figure 1:**
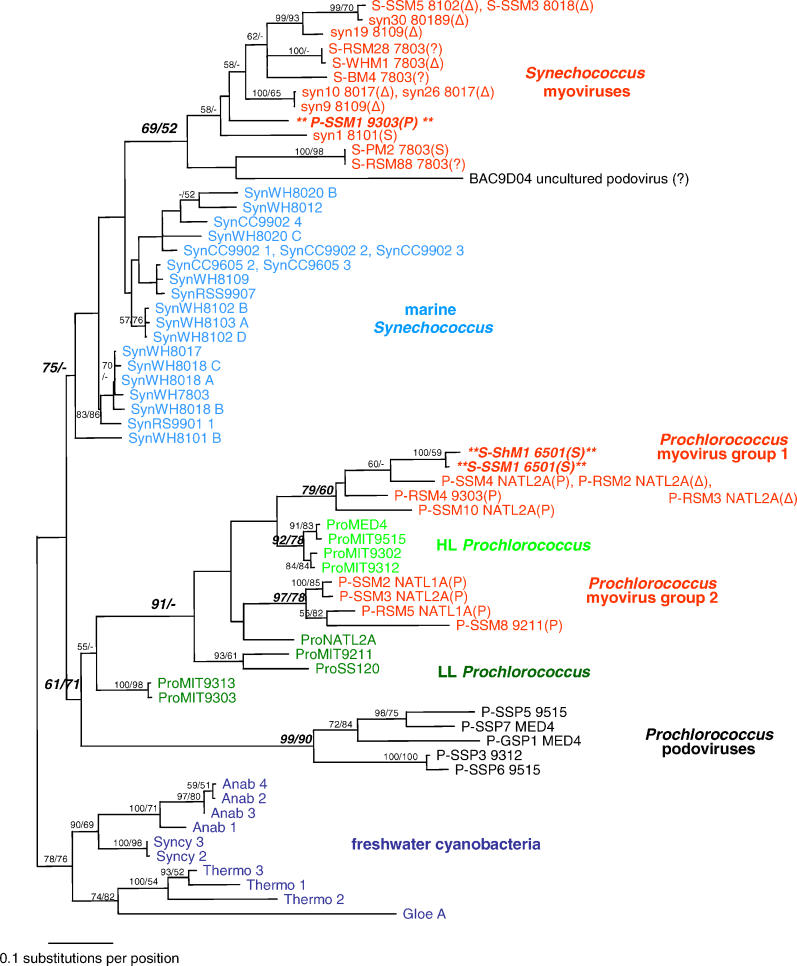
Phylogenetic Tree of *psbA* Gene Sequences from Cultured Cyanobacteria and Cyanophages Phages are listed by their name, followed by their original host. Phages that are known to infect both *Prochlorococcus* and *Synechococcus* hosts are indicated with a “Δ”; those that infect only one genus are labeled either P (infect only *Prochlorococcus* hosts) or S (infect only *Synechococcus* hosts), while those that are unknown are designated with a “?”. Phages shown in italics and bracketed with “**” were isolated on hosts that do not belong to the same cluster and are thus exceptions to the general clustering pattern (see text). Taxa are color coded according to the following biological groupings: myoviruses (red), podoviruses (black), marine *Synechococcus* hosts (light blue), marine *Prochlorococcus* hosts (dark green, LL; light green, HL), freshwater cyanobacteria (dark blue). The tree topology was estimated by LogDet analysis of 1st and 2nd codon positions. Sequences where intragenic recombination was detected using other methods (see Materials and Methods) were not included in these phylogenetic analyses. Branch lengths were estimated by maximum likelihood under a model with nonstationary nucleotide frequencies. Numbers at the nodes represent neighbor-joining bootstrapping and maximum likelihood puzzling support. Anab, *Anabaena;* Gloe, Gleobacter; HL, high-light adapted; LL, low-light adapted; Syncy, *Synechocystis;* Thermo, *Thermosynechococcus*.

**Figure 2:**
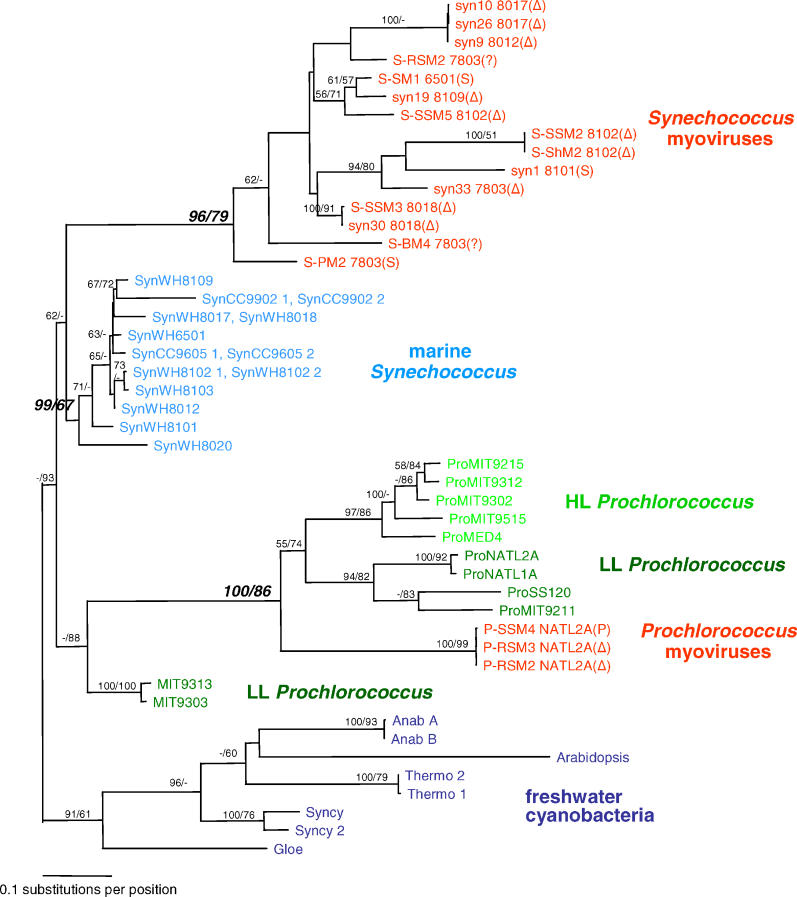
Phylogenetic Tree of *psbD* Gene Sequences from Cultured Cyanobacteria and Cyanophages Details as in Figure 1. Sequences where intragenic recombination was detected using other methods (e.g., P-SSM1) were not included in these phylogenetic analyses.

To investigate the origins of photosynthesis genes in phages and their hosts, we conducted phylogenetic analyses (using measures to minimize systematic errors; see Materials and Methods) of host and phage *psbA* and *psbD* sequences, including new sequence data for nine *Synechococcus* hosts *(psbA),* 19 *Synechococcus* and *Prochlorococcus* hosts *(psbD),* and 33 phages (both *psbA* and *psbD*). Phylogenetic reconstructions of host *psbA* and *psbD* genes in *Prochlorococcus* showed that well-supported sequence clusters contain only one organism type ( Figures 1 and 2), with sequences from high-light adapted (HL) and low-light adapted (LL) *Prochlorococcus* [ 45] forming discrete clusters. These well-supported *Prochlorococcus* clusters are similar to those observed using other host genes such as *rRNA, rpoC1,* and *ntcA* [ 46– 49], indicating that *psbA* and *psbD* have not been transferred between *Prochlorococcus* lineages. In contrast, the *Synechococcus* clusters for both *psbA* and *psbD* are poorly supported, a finding different to that obtained using other highly conserved genes [ 46– 49] and thus may have resulted from genetic exchange between *Synechococcus* lineages.

The *psbA* sequences from *Synechococcus* myoviruses, *Prochlorococcus* myoviruses, and *Prochlorococcus* podoviruses generally formed discrete clusters consistent with their host ranges ( Figure 1), suggesting that the transfer of photosynthesis genes from host to phage has been largely limited by host range (but see exceptions discussed below). Although many of these phages are capable of infecting both host genera (denoted as “Δ” in all figures), we designated each cyanophage isolate as a *Prochlorococcus* or *Synechococcus* cyanophage based upon its original host strain of isolation (as mentioned above and in Table 1). Given this designation scheme, it appears that transfers were predominantly from *Prochlorococcus* to their phages and from *Synechococcus* to their phages. This suggests host-range-limited host-to-phage transfer events, with subsequent horizontal and vertical transfers occurring among viral lineages.

Two isoforms of the PsbA protein are often found in cyanobacteria [ 50]. The PsbA.1 (D1.1) isoform is constitutively expressed, whereas the PsbA.2 (D1.2) isoform is upregulated in response to high light and UV stress [ 51, 52]. Many of the differences between the isoforms are found in ten amino acids between position 121 and 312 [ 50]. Based on which isoform the majority of these ten amino acids were identical to (including glutamine/glutamate at position 130), we determined that PsbA from both *Prochlorocococus* myoviruses and podoviruses are more similar to PsbA.1, the only isoform found in *Prochlorocococus* hosts so far [ 53] (unpublished data). Although *Synechococcus* hosts encode both isoforms (unpublished data), *Synechococcus* myoviruses encode the stress-responsive PsbA.2 isoform exclusively (unpublished data), which may be particularly beneficial during the stress of infection. These findings are consistent with the hypothesis of host-range-limited transfers of the *psbA* gene (but see exceptions below).

Host-to-phage transfers appear to have occurred at least four times for *psbA* and twice for *psbD*, as seen from the number of discrete clades containing phage-encoded genes in each case ( Figures 1 and 2). The four *psbA* gene acquisitions by phage appear to include two transfer events for the *Prochlorococcus* myoviruses ( *Prochlorococcus* myovirus group 1 and 2 in Figure 1) and a single event for *Prochlorococcus* podoviruses all from their *Prochlorococcus* hosts, as well as a single event for *Synechococcus* myoviruses from their hosts ( Figure 1). The *psbD* gene appears to have been acquired once by both *Synechococcus* and *Prochlorococcus* myoviruses from their respective hosts ( Figure 2). Interestingly, the three *Prochlorococcus* myoviruses that contain *psbD* all encode *Prochlorococcus* myovirus group 1 *psbA* sequences, suggesting that this gene was acquired only once by a subset of these myoviruses. Although the specific source is difficult to determine from phylogeny alone, the placement of the *Prochlorococcus* myovirus sequence clusters suggests that *psbA* was derived from either HL *Prochlorococcus* hosts or LL NATL2A-type hosts, while the *psbD* genes could have been acquired from any of the *Prochlorococcus* hosts other than MIT9313/9303. The placement of the *Prochlorococcus* podovirus ( *psbA* only) and *Synechococcus* myovirus sequence clusters at the base of the host and virus clades provides little further information about the source of these phage genes.

We found three exceptions to the above host-constrained evolutionary scenario—i.e., cases where phage *psbA* and *psbD* genes did not cluster with those of their hosts ( Figure 1 and Figures S1 and S2) and did not have PsbA isoforms consistent with that of their hosts (unpublished data). These include two narrow host-range *Synechococcus* myoviruses (S-ShM1, S-SSM1), which encode *psbA* sequences most similar to *Prochlorococcus* myoviruses ( Figure 1) even to the extent that they encode the PsbA.1 isoform, as well as a *Prochlorococcus* myovirus (P-SSM1) with a *psbA* sequence that is most similar to those from *Synechococcus* myoviruses ( Figure 1) and encodes the PsbA.2 isoform as expected for a *Synechococcus* myovirus. Although the latter can cross-infect across *Prochlorococcus* ecotypes, it has not been shown to infect *Synechococcus* [ 5]. The P-SSM1 phage also encodes *psbD,* which, like its *psbA* gene, is more similar to *Synechococcus psbD* sequences than those of the *Prochlorococcus* host upon which it was isolated ( Figure S2; note that this sequence does not appear in Figure 2 because it was a candidate for intragenic recombination; see Materials and Methods). It is likely that these exceptions to the rather consistent host-phage sequence clustering resulted from horizontal transfer events between a broad-host-range donor phage and a limited-host-range recipient phage during coinfection of a single host, i.e., swapping of genes within the phage gene pool [ 24]. Whole gene transfers within the phage gene pool are likely to be more common than this, but undetectable when occurring within phages that form a discrete phylogenetic cluster. These observations call for caution when using clustering patterns of *psbA* and *psbD* sequences from uncultured phage (obtained from environmental genome data) to identify potential hosts.

### Intragenic Recombination within Core Reaction Center Proteins

The lack of well-supported clade structure in phylogenetic reconstructions for *Synechococcus* host strains when using both *psbA* and *psbD* differs from those constructed using other genes [ 46– 49], which led us to wonder about underlying mechanisms that could be responsible for such a blurred phylogenetic signal. In a recent study, Zeidner et al. [ 37] showed that *Synechococcus*-phage-like *psbA* sequences from the environment had a patchy %G+C distribution, which they suggest is due to intragenic recombination [ 37]. Their analyses demonstrated that such recombination had occurred within the inferred-phage clusters and within clusters spanning both phage and host *psbA* sequences. They could not discern, however, whether the signal was caused only by phage-to-phage exchanges, or included phage-to-host exchanges, because the majority of their sequences were of unknown origin (i.e., they were derived from environment clone libraries), and the test employed does not assess the directionality of intragenic recombination events. Our cultured hosts and phages provide an opportunity to assess recombination partners without ambiguity regarding the source of the genes. In addition, the known host ranges of these phages [ 5] ( Table 1), together with the types of recombination tests we have used (see Materials and Methods), allow us to assess the directionality and the pathways through phages and hosts that these recombination events are likely to have taken.

**Figure 3:**
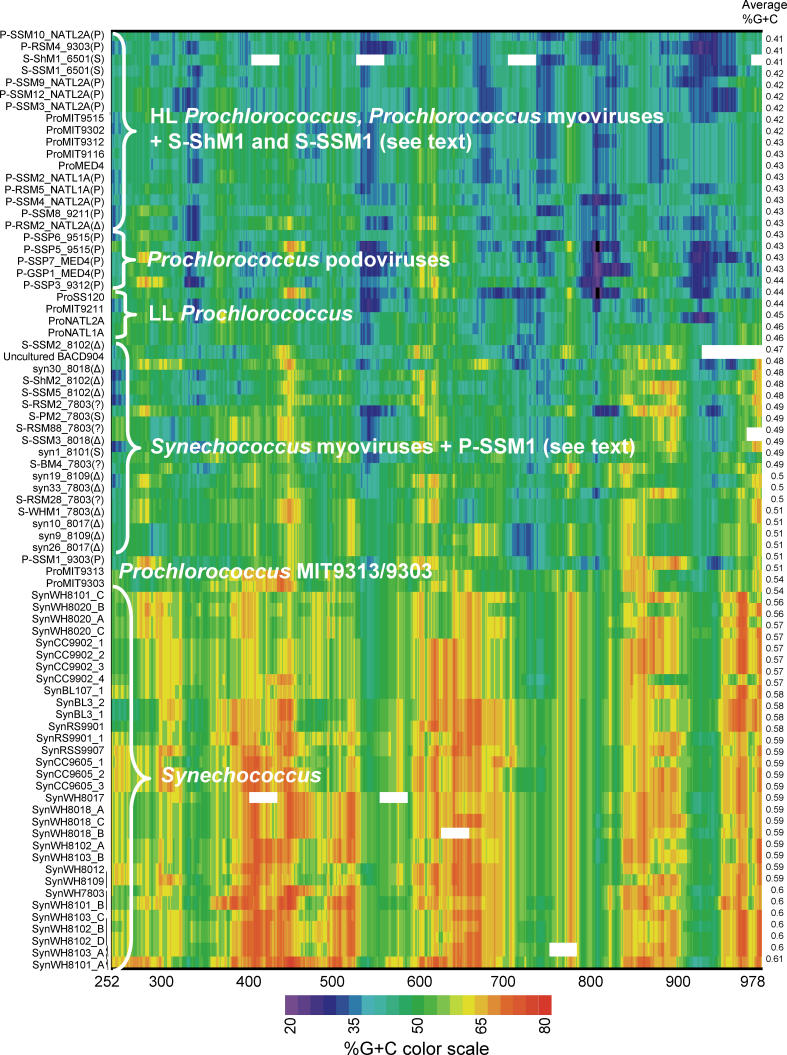
Visualization of %G+C Content across the *psbA* Gene Colors represent the averaged %G+C in sliding windows along the length of the gene (20%–80%); white regions represent windows that included ambiguous bases in which %G+C could not be calculated for that region. The average %G+C content of the amplified sequence is tabulated on the right side of the figure. Phages are listed by phage name followed by their original host. Phages that are known to infect both *Prochlorococcus* and *Synechococcus* hosts are indicated with a “Δ”; those that infect only one genus or the other have no marker, while those that are unknown are designated with a “?”. Host names are prefaced with Syn or Pro for *Synechococcus* and *Prochlorococcus* hosts, respectively. Scale indicates nucleotide positions relative to the *psbA* gene sequence in *Thermosynechococcus*.

**Figure 4:**
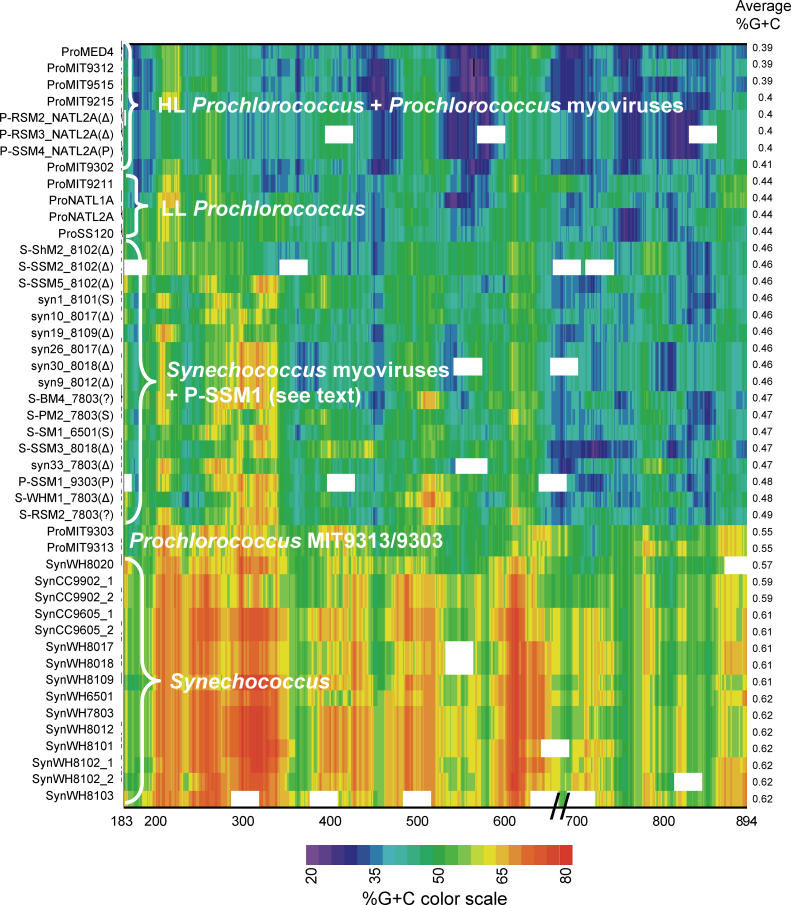
Visualization of %G+C Content across the *psbD* Gene Details as in Figure 3. Note that the 21-nucleotide indel in *Prochlorococcus* hosts and their phages [ 13] (unpublished data) was excluded from the analysis at the position indicated by the “//” symbol to maximize the data that could be displayed using the sliding window approach.

As a first assessment for potential intragenic recombination, we analyzed the %G+C patterns in all of the *psbA* and *psbD* genes ( Figures 3 and 4, respectively). *Prochlorococcus* phage genes had similar average %G+C contents to those from their *Prochlorococcus* hosts (39%–46%), whereas those of *Synechococcus* phages had %G+C contents that were lower than those from their *Synechococcus* hosts (46%–51% versus 56%–62%), but not as low as those from *Prochlorococcus* hosts and phages *.* This intermediate %G+C could be the result of intragenic recombination between variants of the two host lineages. Alternatively, it may reflect the current state of mutational amelioration of the acquired gene from a high %G+C source towards the low genome-wide %G+C of the virus ( *Synechococcus* myoviruses S-PM2 and Syn9 both have low genome-wide %G+C; [ 28]; P. Weigele, W. Pope, G. Hatfull, R. Hendrix, personal communication). If the latter is the case, we might expect such amelioration to be constant across the gene, resulting in an even %G+C distribution pattern.

To help differentiate between these hypotheses, we mapped the %G+C variation across the *psbA* and *psbD* genes using the methodology developed by Zeidner et al. [ 37]. We detected patchiness of %G+C in *Synechococcus* myovirus *psbA* sequences dispersed along the length of the gene ( Figure 3), confirming the findings reported by Zeidner et al. [ 37]. We also detected %G+C patchiness among *psbA* from *Prochlorococcus* podoviruses, but not from *Prochlorococcus* myoviruses, despite overall similarity of their %G+C content with their *Prochlorococcus* hosts. This suggests that intragenic recombination has occurred among the podoviruses *.* In addition, patterns of %G+C were not uniform and even markedly clumped across the *psbD* gene from *Synechococcus* myoviruses ( Figure 4), with the first segment resembling *Synechococcus* hosts and the last segment resembling *Prochlorococcus* hosts and their phages. Thus, intragenic recombination is likely to be at least partly responsible for the intermediate %G+C content in *Synechococcus* myovirus *psbA* and *psbD* sequences.

Statistical methods for detecting intragenic recombination (see Materials and Methods) revealed strong evidence for its presence in both the *psbA* and *psbD* sequence sets ( Tables S1 and S2), but the relative frequency of recombination events was not equal for different groups of hosts and phages. Recombination appears most common among the cyanophages, and more so for *Synechococcus* than *Prochlorococcus* phages. Exchanges were detected between phages that infect both *Synechococcus* and *Prochlorococcus* as well as within myoviruses that infect a single genus *(Synechococcus).* Note that exchanges within a single phylogenetic phage cluster, such as within the *Synechococcus* myoviruses, were undetectable by our previous phylogenetic analyses. Interestingly, our analyses also revealed exchanges between *Prochlorococcus-*specific podoviruses and broad-host-range *Synechococcus* myoviruses, with the *Prochlorococcus* podoviruses serving as the donors ( Table S1). Marine cyanobacterial podoviruses contain integrase genes and are thought to have the ability to integrate into the genomes of their hosts as prophages [ 30] (P. Weigele, W. Pope, G. Hatfull, R. Hendrix, personal communication). If true, genetic exchange could occur between the *Prochlorococcus* prophage and a *Synechococcus* lytic phage—a scenario well accepted in other phage-host systems for genetic exchange [ 14, 15].

Intragenic recombination involving host genes appears less common than phage-to-phage recombination events ( Tables S1 and S2). Exchanges between *Synechococcus* and their viruses are evident, however, and appear to have occurred both from host to phage and phage to host for both *psbA* and *psbD*. Although such events were not detected between *Prochlorococcus* and their phages, there were cases where *Prochlorococcus* myoviruses were the recipients of external DNA from an unknown source (i.e., recombination events possibly involving donors outside of our dataset). Thus, phages may be contributing to the intragenic recombination of portions of these genes in *Synechococcus,* perhaps explaining the lack of phylogenetic structure observed in *psbA* and *psbD* trees for *Synechococcus* clusters (but not for *Prochlorococcus* clusters) relative to those obtained when using other phylogenetic markers [ 46– 49]. Presumably, phage-host intragenic exchanges occur via homologous recombination during infection. Clearly, the transfer of DNA will be retained in host lineages only if infection fails to lyse the host (e.g., abortive infection [ 54]).

Finally, intragenic exchanges among hosts were also occasionally detected, particularly among *Synechococcus* ( Tables S1 and S2). This may also play a role in the lack of clade structure among *Synechococcus* strains in the *psbA* and *psbD* trees. Although two possible intragenic recombination events between *Synechococcus* and *Prochlorocococus* were identified, they were resolved as small regions (15–16 bases) and may be false positives. Host-to-host transfers may have occurred through the uptake of DNA directly from the environment (e.g., via transformation) or through viral intermediates [ 37]. Such host-to-host intragenic exchanges via viral intermediates presumably occur through generalized transduction [ 55].

In summary, our findings suggest that the shuffling of segments of *psbA* and *psbD* within the cyanophage gene pool has generated significant photosynthesis gene diversity and serves as an extended reservoir of genetic diversity for their hosts, influencing photosystem evolution.

### *psbA* and *psbD* Gene Diversity in Cultured Isolates Captures Most of the Field Diversity

**Figure 5:**
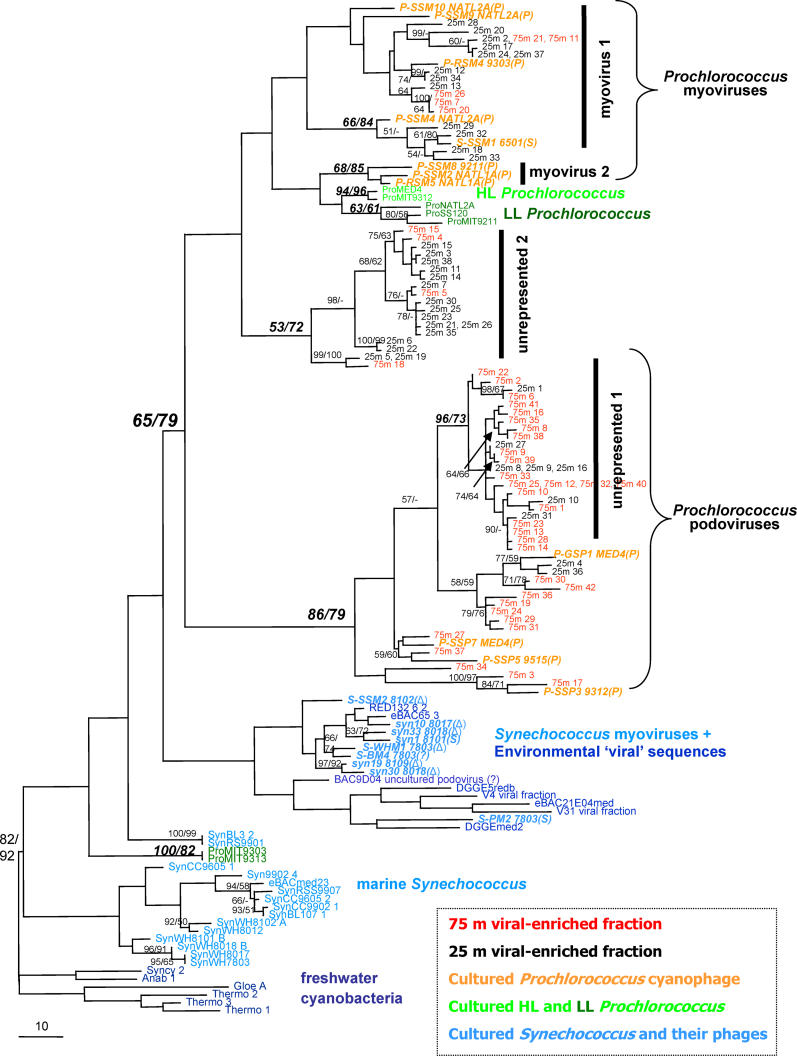
Phylogenetic Tree of *psbA* Gene Sequences from Representative Cultured Cyanobacterial and Cyanophage Isolates and Cloned Environmental Sequences from the Hawaii Ocean Time Series Site in the Pacific Ocean Phylogenetic tree of *psbA* gene sequences and cloned environmental sequences were collected from above (25 m, black) and below (75 m, red) the surface mixed layer at the Hawaii Ocean Time Series site in the Pacific Ocean, a region where *Prochlorococcus* are the dominant phototrophs. Details for naming conventions are as in Figure 1. *Synechococcus* environmental “viral” sequences from [ 37]. The tree topology was estimated by LogDet analysis of 1st and 2nd codon positions, with branch lengths estimated using stationary nucleotide frequencies.

**Figure 6:**
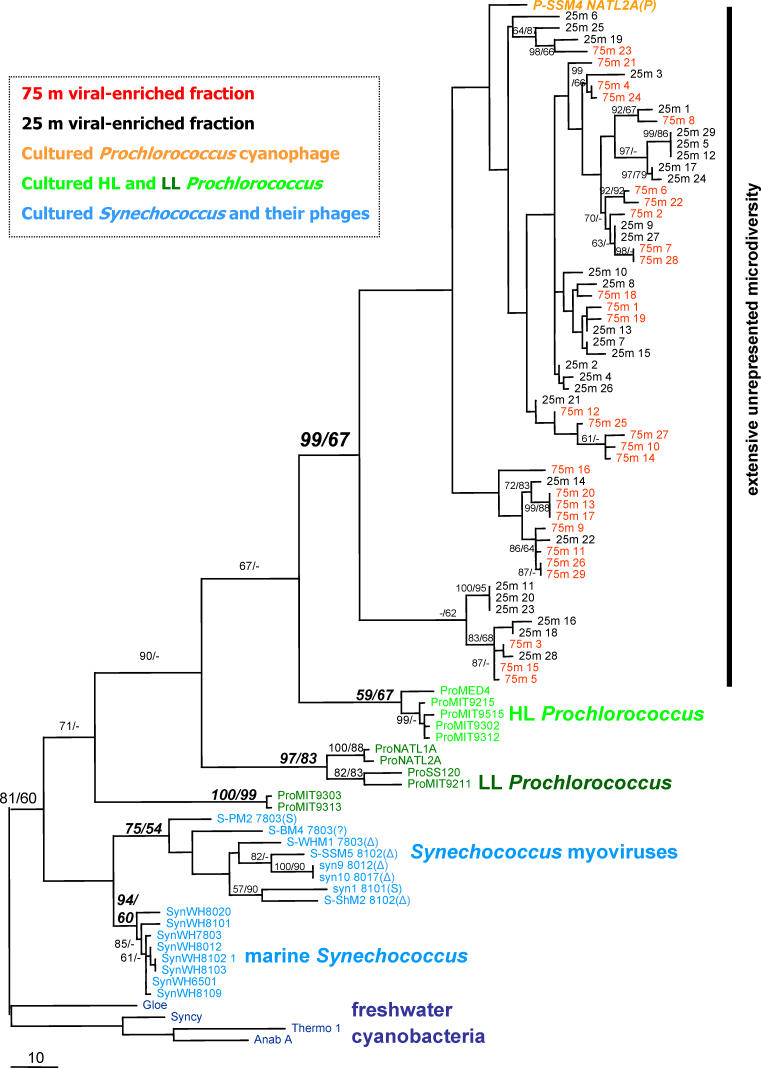
Phylogenetic Tree of *psbD* Gene Sequences from Cultured Cyanobacterial and Cyanophage Representatives and Cloned Environmental Sequences from the Pacific Ocean Details for naming conventions are as in Figure 1, and phylogenetic analyses are as in Figure 5.

We next sought to determine how well *psbA* and *psbD* sequence diversity observed in culture collections represents that observed in wild phage populations, and whether additional whole-gene host-to-phage transfer events could be identified from these wild sequences from the phage gene pool. Zeidner et al. [ 37] had previously examined field diversity of the *psbA* gene sequence from environmental samples where *Synechococcus* strains were the dominant phototroph [ 37]. Thus, we sought to examine genetic diversity of this gene, as well as that of *psbD,* from an environment where *Prochlorococcus* cells commonly outnumber *Synechococcus* cells by orders of magnitude [ 56]. To this end, we amplified, cloned, and sequenced *psbA* and *psbD* gene sequences obtained from the viral-sized fraction (0.02–0.2 μm) of two seawater samples within (25 m) and below (75 m) the mixed layer in the Pacific Ocean off the coast of Hawaii ( Figures 5 and 6, respectively). The *psbA* and *psbD* sequences from these viral-fraction samples clustered with cultured *Prochlorococcus* cyanophage isolates (with varying levels of support; Figures 5 and 6), but not with *Synechococcus* cyanophages. There was not a notable difference in the phylogenetic placement of the *psbA* or *psbD* clones obtained from within or below the mixed layer. Although this suggests a lack of vertical structure in diversity among the sequence types, we did not sequence these samples to saturation; thus, such conclusions are preliminary.

More than half of the wild *psbA* sequences (42 of 81) form a large cluster with cultured *Prochlorococcus* podoviruses ( Figure 5). Within this group, all but one cluster of wild sequences contain cultured podovirus sequences ( Figure 5). The extensive microdiversity in this cluster (labeled “unrepresented 1”) was probably derived from within the podovirus gene pool, as evidenced by the presence of podovirus phage isolates in the more basal branches of the cluster. Other *psbA* sequences from the field samples form subclusters that contain cultured *Prochlorococcus* myoviruses and form a large group that also contains *Prochlorococcus* hosts ( Figure 5). One cluster (“unrepresented 2” in Figure 5) within this group also lacks sequences from cultured hosts or phages. The basal position of this cluster suggests that these sequences may belong to phages that infect as-yet uncultured *Prochlorococcus* hosts [ 57] and may represent an additional host-to-phage transfer event. Thus, our work here, together with that of Zeidner et al. [ 37], suggests that cyanophage culture collections represent much of the naturally occurring *Prochlorococcus* and *Synechococcus* cyanophage *psbA* gene sequence diversity [ 37].

All *psbD* sequences from wild phages fall into a single well-supported cluster that includes a representative cultured *Prochlorococcus* cyanophage P-SSM4 ( Figure 6). This cluster reveals significant microdiversity within the *psbD Prochlorococcus* phage gene pool in the viral-fraction from this Pacific Ocean site and suggests that phages that encode *Prochlorococcus*-phage-like *psbD* genes are perhaps not rare in this environment. The four *Prochlorococcus* cyanophages that contain the *psbD* gene in our culture collection originated from either the Sargasso Sea or the Red Sea; thus, it is perhaps not surprising that the viral-fraction microdiversity from the Pacific Ocean is largely unrepresented in this collection.

### Conclusions

The phage genomic repertoire evolves through the exchange of genetic material from other phages [ 24] and by co-opting metabolic genes from their hosts [ 13, 20, 22]. The prevalence of photosynthesis genes in cyanophages strongly suggests that the capture of these genes provides a significant fitness advantage among certain cyanophage types. Previously, we have shown that the horizontal transfer of *hli* genes from cyanophages to their hosts has likely played a role in driving host niche differentiation [ 13]. More recently, cyanophages were hypothesized to be involved in partial gene exchanges even for the core photosystem gene *psbA* of their hosts [ 37]. Here, we show that genetic exchanges involving cyanophages may have influenced the make-up of both of the core photosystem II genes *(psbA* and *psbD)* in *Synechococcus,* whereas this was less apparent for *Prochlorococcus*. Therefore, mounting evidence indicates that host-like genes acquired by phages undergo a period of diversification in phage genomes and serve as a genetic reservoir for their hosts. Thus, a complex picture of overlapping phage and host gene pools emerges, where genetic exchange across these pools leads to evolutionary change for host and phage. Fully understanding the mechanisms of microbial and phage coevolution clearly requires an improvement in our ability to quantify horizontal gene transfer at the whole and partial gene level and in our ability to accurately estimate the relative fluxes into and out of these pools.

## Materials and Methods

### DNA isolation from cultured hosts and phages and environmental samples.

Eleven strains of *Prochlorococcus,* ten strains of *Synechococcus,* and 38 phages of *Prochlorococcus* and *Synechococcus* (seven podoviruses, 29 myoviruses, and two siphoviruses) were screened for *psbA* and *psbD* sequences for this study. We report here on new *psbA* sequences from nine *Synechococcus* hosts and new *psbD* sequences from 19 *Prochlorococcus* and *Synechococcus* hosts (including two from unpublished *Synechococcus* genomes for strains CC9605 and CC9902; available from http://genome.jgi-psf.org/mic_home.html. The 38 phages screened included seven phage templates for which genome sequences are now available (P-SSM2, P-SSM4, P-SSP7, S-PM2, S-WHM1, Syn5, Syn9), enabling us to validate our PCR amplification findings. Host genomic DNA was extracted using a DNeasy Tissue Kit (Qiagen, Valencia, California, United States). Filtered (0.2 μm, Acrodisc supor membrane syringe filter) phage lysates in Pro99 medium were used as DNA templates for subsequent PCR amplification experiments.

Environmental samples were collected from the Hawaii Ocean Time Series (HOT) on 15 October 2003 at 45°N 158°W from depths of 25 m and 75 m. These samples were filtered through a 0.2-μm filter (Osmonics, Minnetonka, Minnesota, United States, Poretics polycarbonate 25-mm filter) to remove cellular material and substantially enrich for environmental phages. A 100-ml volume of 0.2-μm filtrate was then filtered onto a 0.02-μm filter (Whatman Anotop 25) to collect phage particles and resuspended in 7 ml of a modified SM storage buffer (600 mM NaCl, 8 mM MgSO _4_-7H _2_O, 50mM Tris [pH 7.5], 0.04% gelatin).

### Overview of *psbA* and *psbD* screening strategy.

PCR screening for *psbA* and *psbD* across a diverse set of samples presented several challenges. These included variable amplification efficiencies, uncertainty about whether amplicons derived from phage or host, and multiple gene copies in hosts. The amplification strategy was as follows: for each virus and host strain, four PCR reactions were carried out, pooled, and analyzed by gel electrophoresis; if the amplification product was not visible, it was diluted 10-fold and used as template for nested or semi-nested PCR and the resulting products analyzed; if still no product was visible, multiple phage stocks were rescreened. Multiple copies of *psbA* in *Synechococcus* strains were identified by sequencing many clones and were distinguished from sequencing errors as described below. We did not screen for multiple copies of *psbA* from *Prochlorococcus* or multiple copies of *psbD* from either *Synechococcus* or *Prochlorococcus,* as when present, they are generally indistinguishable from each other [ 58– 60].

### Amplification of *psbA* and *psbD*

PCR reactions were performed with *Taq* DNA polymerase and deoxyribonucleotide triphosphates from New England Biolabs (Beverly, Massachusetts, United States) or Invitrogen (Carlsbad, California, United States) and carried out with a PTC-100 or PTC-200 DNA Engine (MJ Research, Waltham, Massachusetts, United States) or a Robocycler Gradient 96 (Stratagene, La Jolla, California, United States). Template amounts were 10 ng of genomic DNA for *Prochlorococcus* and *Synechococcus,* 1 μl of lysate for cyanophages, and 2 μl of filtrate for environmental samples. PCR primers and amplification reaction conditions are shown in Tables S3 and S4.

The *psbA* gene from all sources was amplified using primer pair *psbA*-F/R [ 61] and PCR protocol A ( Tables S3 and S4). Four reactions were conducted with each template, and the products were pooled and analyzed by agarose gel electrophoresis. Primer *psbA*-R falls on the intron region in S-PM2 [ 29]. Therefore, for efficient amplification of phage *psbA* genes that may contain introns, and for increased sensitivity, we used the Pro- *psbA*-F/R primer set and protocol B in nested PCR reactions when no PCR product was visible from cyanophage lysates and environmental filtrates. To reduce the incidence of heteroduplex formation, amplification products from environmental samples were subjected to reconditioning PCR [ 62]: initial PCR products were diluted 1:10, then amplified using protocol A but for only three cycles.

The *psbD* gene from *Prochlorococcus, Synechococcus,* and cyanophages was amplified using primer pair *psbD-*54F/ *psbD*-308R and protocol D. However, when product yield was low or absent, semi-nested PCR was carried out as follows. Amplification was first conducted using primer pair *psbD*-26F/ *psbD*-308R and protocol C. Four reactions were conducted with each template, the products were pooled, diluted 1:10, and used as templates for a second round of amplification using primer pair *psbD*-54F/ *psbD*-308R and protocol D. *psbD* from environmental samples was amplified using primer pair *psbD*-26F/ *psbD*-308R and protocol C and subjected to reconditioning PCR as for *psbA* (see above).

In preparation for sequencing, PCR products were either purified directly using the QIAquick PCR Purification Kit (Qiagen) or separated on an agarose gel and then purified using the QIAquick Gel Extraction Kit (Qiagen).

To confirm that the absence of *psbA* or *psbD* PCR products from phage was not simply due to a lack of amplifiable phage DNA, we screened phage lysates for known phage genes: *g20* (for myoviruses) and *DNApol* (for podoviruses). *g20* was amplified using primer pair g20-F/R and protocol E, and *DNApol* using primer pair DNApol-F/R and protocol F, both with 1 μl of lysate. In all cases, a product was obtained, suggesting the phage template DNA was present and amplifiable by PCR (unpublished data).

Six phage lysates yielded PCR products with sequences identical to those of a known host. These six phage lysates include five cyanophages previously described (P-RSP1, P-SSP1, P-SSP2, P-ShM1, P-ShM2; [ 5]), as well as one cyanophage not previously reported in the literature (P-SSP9; M.B.S. and S.W.C., unpublished data). In these cases we could not eliminate the possibility that the amplicon resulted from host DNA, the amplification of which may be more likely to occur when there is no phage template for this gene. Thus, we excluded these phages from further analyses. In contrast, phages with amplicon sequences identical to those of other phages (indicated as “ID to X” in Table 1) were passed through multiple lysates, and a “fingerprint” phage gene *(g20)* was used to confirm that there was a single phage in the lysate. The *psbA* sequence was then re-assayed, increasing our confidence in these results. Even with this precaution, we cannot rule out the possibility of PCR contamination for those few cases where identical sequences were amplified from different phage lysates.

### Cloning and sequencing of PCR products.

The *psbA* gene is often found in multiple distinct copies in marine *Synechococcus* [ 59], whereas in *Prochlorococcus* the *psbA* gene is either single copy per genome or encodes multiple copies that are nearly identical to each other [ 60, 63, 64]. Among cyanophages, the *psbA* gene has only been found in a single copy per genome [ 28, 30]. To allow for the identification of multiple *psbA* gene copies in *Synechococcus* strains, PCR products from *Synechococcus* templates were cloned prior to sequencing. Cloning was performed using the TOPO TA Cloning Kit for Sequencing (Invitrogen) with the pCR4-TOPO vector. Ligation products were transformed into TOP10 competent cells. Plasmid purification and sequencing were conducted by Genaissance Pharmaceuticals (New Haven, Connecticut, United States). Inserts were sequenced from both forward and reverse directions, using the M13F and M13R primer binding sites in the pCR4-TOPO vector.

Approximately ten *psbA* clones were sequenced for each *Synechococcus* strain. The published genome of *Synechococcus* WH8102 provides an example of natural *psbA* diversity in a given strain, as it contains four copies of *psbA:* two copies that are 99.8% identical and a third and fourth copy that are 99.4% and 88% identical, respectively, to the above two *psbA* copies [ 59]. Considering a *Taq* polymerase error rate of 3 × 10 ^−5^ per nucleotide per duplication [ 65], at most one error could be expected in each *psbA* gene sequenced. Thus, sequences were considered identical, and removed from the analysis pool, if they were more than 99.8% identical, to avoid data issues stemming from possible PCR error (sequencing error should be nonexistent because consensus sequences were obtained from forward and reverse sequencing of the clones). Sequence identity levels for nonidentical clones from the remaining dataset ranged from about 60% to 99.0%.

PCR products from genes presumed not to have multiple distinct copies per genome ( *psbA* from *Prochlorococcus* and cyanophage; *psbD* from all organisms) were generally sequenced directly (Harvard Medical School Biopolymers Facility [Boston, Massachusetts, United States], Davis Sequencing [Davis, California, United States], or Genaissance Pharmaceuticals). The absence of multiple significant-height peaks at single nucleotide positions in chromatograms from this direct sequencing (unpublished data) confirmed that single products were amplified during PCR. Each strain was sequenced in both forward and reverse directions, using the same primers used for PCR amplification.

### Sequence analyses.

Previous analyses have raised important concerns about using *psbA* gene sequence datasets that may suffer from large %G+C variability and conflicting phylogenetic signals in phylogenetic reconstructions [ 37]. To minimize such errors, we followed these steps.

We first performed phylogenetic analyses using sequences from all taxa (80 for *psbA* and 50 for *psbD*) and all codon positions ( Figures S1 and S2). Phylogenetic trees were constructed by using distance and maximum likelihood. Neighbor-joining [ 66] was used to reconstruct a distance tree under the HKY85 model [ 67]. Maximum likelihood analysis was performed under HKY85 combined with a gamma model for among sites rate variation, assuming eight rate categories with model parameters estimated from the data [ 68]. Maximum likelihood trees were obtained by quartet puzzling, as implemented in the program TREE-PUZZLE 5.0 [ 69]. Bootstrap resampling (1,000 pseudoreplicates) was used to measure the relative support for internal branches of the neighbor-joining trees. For quartet puzzling, support was estimated from 25,000 ( *psbD* trees) or 50,000 ( *psbA* trees) pseudoreplicates.

These analyses resulted in trees with high bootstrap support at many critical nodes ( Figures S1 and S2). However, fitting a single tree to large datasets containing conflicting phylogenetic signals can lead to reconstruction artifacts (i.e., systematic errors) that result in high bootstrap support [ 70, 71]. We found, using neighbor-nets [ 72] constructed by using the SplitsTree2 program [ 73], within-gene conflicting phylogenetic signals in both the *psbA* and *psbD* datasets as indicated by the box-like structures in neighbor-nets graphs ( Figures S3 and S4). Specifically, networks for both genes revealed substantial conflict involving splits between *Synechococcus* strains, their myoviruses, and a complex of sequences comprised of *Prochlorococcus* and their viruses.

We further investigated whether these large datasets could suffer from systematic errors related to: (i) substitution rate variation among lineages [ 74], (ii) heterogeneous compositional bias among lineages (e.g., %G+C; [ 75]), and (iii) within-gene heterogeneity in phylogenetic signals [ 76]. We found significant substitution rate variation among lineages ( Table S5) using likelihood ratio tests. In addition, nucleotide frequencies were nonstationary across these data, with significant differences in equilibrium frequencies for clades defined according to organism types ( Table S6; [ 77]). Not surprisingly, the largest divergence in %G+C across taxa was at the 3rd codon positions of both *psbA* and *psbD*.

Zeidner et al. [ 37] hypothesized intragenic recombination in *psbA* [ 37]. We attempted to identify this qualitatively through graphical analysis of %G+C and quantitatively using four different tests for intragenic recombination. The %G+C distribution was examined within overlapping sequence windows (a sliding window of 30 nucleotides with a five-nucleotide step) using the GCViz script [ 37] (available upon request from Dr. Shmoish of Technion–IIT; E-mail: mshmoish@cs.technion.ac.il) written in the R-language ( http://www.r-project.org). Three of the four different tests for within-gene recombination are based on the distribution of substitutions ( GeneConv: [ 78]; MaxChi: [ 79]; Chimaera: [ 80]), while the fourth used a phylogenetic approach (“RDP,” as implemented in [ 81]). We considered only those recombination events that satisfied all of the following criteria: (i) results were significant after application of Bonferroni correction for multiple tests, (ii) regions were detected by two or more different methods, and (iii) consensus breakpoints could be estimated for a given region identified using different methods. Once a putative recombination event was detected, we inferred the best candidate donor sequence (that most similar to the recombinant segment) using RDP [ 81].

In summary, to minimize systematic errors in the ultimate phylogenetic analyses, we first processed the dataset as follows: (i) excluded those sequences having a strong signal for intragenic recombination, (ii) excluded 3rd codon positions, which display the largest differences in %G+C and substitution rates among lineages, and (iii) employed LogDet distances [ 75] to accommodate compositional heterogeneity (variable %G+C) in the remaining data. These measures proved to be important. The uncorrected dataset grouped lineages according to evolutionary rates and %G+C bias ( Figures S1 and S2), whereas the ultimate analysis did not (see Figures 1 and 2). Statistical analysis of the processed dataset under nonhomogenous evolutionary models [ 77] revealed that the ultimate phylogenetic hypotheses (see Figures 1 and 2) provided a significantly better fit to the data ( Table S7). Prior to processing the data, the alternative phylogenies were indistinguishable ( Table S7).

## Supporting Information

Figure S1 Phylogenetic Analyses Including All *psbA* Gene Sequences from Cultured Cyanobacteria and Cyanophages
Phages are listed by phage name, followed by their original host. Host range information is designated in parentheses. Phages known to infect both *Prochlorococcus* and *Synechococcus* hosts are indicated with a “Δ”; phages that infect only *Prochlorococcus* or *Synechcococcus* are designated by a P or S, respectively; and those host ranges that are unknown have a “?”. Phages shown in italics and bracketed with “**” were isolated on hosts that do not belong to the same cluster and are thus exceptions to the general clustering pattern (see text). Taxa are color coded according to the following biological groupings: myoviruses (red), podoviruses (black), marine *Synechococcus* hosts (light blue), marine *Prochlorococcus* hosts (dark green, HL; light green, LL), freshwater cyanobacteria (dark blue). Neighbor-joining tree was inferred under HKY85 mode and using sequences from all taxa and all codon positions. Nucleotide frequencies were assumed to be homogenous across lineages. Numbers at the nodes represent neighbor-joining bootstrapping and maximum likelihood puzzling support. Anab, *Anabaena;* Gloe, Gleobacter; HL, high-light adapted; LL, low-light adapted; Syncy, *Synechocystis;* Thermo, *Thermosynechococcus*.
(79 KB PPT)

Figure S2 Phylogenetic Analyses Including All *psbD* Gene Sequences from Cultured Cyanobacteria and Cyanophages
Details are as in Figure S1.
(59 KB PPT)

Figure S3 Neighbor-Nets Analysis of 80 *psbA* Gene Sequences (including All Cyanophage and Marine Cyanobacterial Sequences Available)
The analysis was conducted under the HKY85 model of substitution using all codon positions. Taxa color coding and abbreviations are as in Figure S1. The box-like appearance in the basal branches of this phylogeny suggests regions of conflicting phylogenetic signals (see Materials and Methods).
(272 KB PDF)

Figure S4 Neighbor-Nets Analysis of 50 *psbD* Gene Sequences (including All Cyanophage and Marine Cyanobacterial Sequences Available)
Taxa color coding and abbreviations are as in Figure S1. Details of the analysis are as in Figure S3.
(249 KB PDF)

Table S1 Consensus Results from Four Tests for Intragenic Recombination within Gene Sequences in Our *psbA* Dataset
The four tests included (1) RDP, (2) GeneConv, (3) MaxChi, and (4) Chimaera (as described in Materials and Methods), and recombination was considered “detected” only when the following criteria were satisfied: (i) similar regions were detected by two or more methods, (ii) all such regions were significant at *p* < 0.05 after a Bonferroni correction for multiple tests, and (iii) consensus breakpoints could be inferred from the results. Thus, “No recombination detected” does not preclude that intragenic recombination could be occurring within the sequence, but rather indicates that our stringent criteria have not identified such an event. While we define phages as either *Prochlorococcus* or *Synechococcus* phages depending on the original host of isolation, we note that many of the myoviruses cross-infect both genera (represented with a “Δ” where known, a “?” where unknown, and no symbol for isolates that do not cross-infect across genera). Consensus breakpoints are relative to nucleotide positions in *Thermosynechococcus psbA.*
(29 KB XLS)

Table S2 Consensus Results from Four Tests for Intragenic Recombination within Gene Sequences in Our *psbD* Dataset
Details are as in Table S1.
(28 KB XLS)

Table S3 PCR Conditions (38 KB DOC)

Table S4 PCR Primers (39 KB DOC)

Table S5 Likelihood Ratio Tests for Variable Evolutionary Rates among Branches For both *psbA* and *psbD,* individual sequences exhibiting a signature for intragenic recombination ( Tables S1 and S2) were excluded from analysis. Likelihood scores were obtained under a stationary HKY85 model combined with a gamma correction for among-sites rate variation. All model parameters, including nucleotide frequencies, were estimated by using maximum likelihood. Data analysis included all three codon positions. Models were employed as implemented in the baseml program of the PAML package [ 82]. Tree 1 was obtained by neighbor-joining analysis of LogDet distances estimated from all three codon positions. Tree 2 was obtained by neighbor-joining analysis of LogDet distances estimated from 1st and 2nd codon positions. For both genes, Tree 1 grouped lineages along lines of similarity in evolutionary rates and compositional biases, and Tree 2 did not.
(36 KB DOC)

Table S6 Likelihood Ratio Tests for Nonstationary Frequencies among Lineages H _0_ denotes the null hypothesis of stationary nucleotide frequencies; this was modeled by specifying one set of nucleotide frequencies for all branches of the tree. H _1_ denotes the alternative hypothesis of nonstationary nucleotide frequencies; this was modeled by assigning all branches of the tree topology to one of several independent sets of frequency parameters (six sets for *psbA* and five sets for *psbD*). Apart from nucleotide frequencies, H _0_ and H _1_ assumed a substitution process equivalent to an HKY85 model combined with a gamma model for among-sites rate variation. The transition/transversion ratio was assumed to be homogenous among branches. H _1_ represents a user-defined version of the nonhomogenous models of Yang and Roberts [ 77]. All model parameters, including nucleotide frequencies, were estimated by using maximum likelihood. Data analysis included all three codon positions. Models were employed as implemented in the baseml program of the PAML package [ 82].
Tree 1 was obtained by neighbor-joining analysis of LogDet distances estimated from all three codon positions. Tree 2 was obtained by neighbor-joining analysis of LogDet distances estimated from 1st and 2nd codon positions. For both genes, Tree 1 grouped lineages along lines of similarity in evolutionary rates and compositional biases, and Tree 2 did not. User-defined sets of frequency parameters for H _1_ were specified in the tree file (shown below) by using the “branch label” format described in the PAML manual. For both *psbA* and *psbD,* individual sequences exhibiting a signature for intragenic recombination ( Tables S1 and S2) were excluded from analysis.
(44 KB DOC)

Table S7 Likelihood-Based Statistical Comparison of Competing Evolutionary Hypotheses under a Model of Nonstationary Nucleotide Frequencies *P*_KH_ denotes the *p*-value for the KH normal test of [ 83]. *P*_SH_ denotes the *p*-value for the SH test [ 84]. *P*_RELL_ denotes the RELL bootstrap proportion [ 83]. Note that although Tree 1 and Tree 2 were not selected independently of the data, neither was selected according to its likelihood score. For both genes, Tree 1 grouped lineages along lines of similarity in evolutionary rates and compositional biases, and Tree 2 did not. For both *psbA* and *psbD,* individual sequences exhibiting a signature for intragenic recombination ( Tables S1 and S2) were excluded from analysis. Tree 1 was estimated by a neighbor-joining analysis of LogDet distances from all sites, and Tree 2 was estimated by a neighbor-joining analysis of LogDet distances based on only 1st and 2nd codon positions. Likelihood scores were obtained under nonstationary models of nucleotide frequencies (see Table S5 for additional model details).
(46 KB DOC)

### Accession Numbers

New sequences from cultured cyanobacteria and cyanophages are deposited in GenBank ( http://www.ncbi.nlm.nih.gov/Genbank) under accession numbers DQ473647–DQ473719, whereas new environmental sequences are deposited under accession numbers DQ473720–DQ473847.

## Abbreviations

HL: high-light adapted
LL: low-light adapted
