# Supplementary material for: Prevalence and Evolution of Core Photosystem II Genes in Marine Cyanobacterial Viruses and Their Hosts
Source: PLoS Biol. 2006 Jul 4;4(8):e234. doi: 10.1371/journal.pbio.0040234 (PMC1484495; doi:10.1371/journal.pbio.0040234)
Supplement: Supplementary file 3 [file pbio.0040234.st003.doc]

**Supplementary Table 3**. PCR conditions

| **PCR Protocol** | **Forward Primer** | **Reverse Primer** | **Primer Conc. (µ*M*)** | **dNTP Conc. (µ*M*)** | **MgCl_2_ Conc. (m*M*)** | **Units of *Taq*** | **Reaction Volume (µL)** | **Initial Denaturation** | **# of Cycles** | **Cycled Denaturation** | **Cycled Annealing** | **Cycled Extension** | **Final Extension** |
| --- | --- | --- | --- | --- | --- | --- | --- | --- | --- | --- | --- | --- | --- |
| A | *psbA*-F | *psbA*-R | 0.25 | 200 | 2.5 | 2.0 | 20.0 | 94°C, 5 min | 35 | 94°C, 1 min | 52°C, 1 min | 72°C,  1.5 min | 72°C, 10 min |
| B | Pro-*psbA*-F | Pro-*psbA*-R | 0.25 | 80 | 5.0 | 2.5 | 50.0 | 92°C, 4 min | 35 | 92°C, 1 min | 50°C, 1 min | 68°C, 1 min | 68°C, 10 min |
| C | *psbD*-26F | *psbD*-308R | 1.00 | 200 | 1.5 | 1.0 | 20.0 | 94°C, 5 min | 35 | 94°C, 1 min | 51°C, 1 min | 72°C, 1 min | 72°C, 10 min |
| D | *psbD*-54F | *psbD*-308R | 1.00 | 200 | 1.5 | 1.0 | 20.0 | 94°C, 5 min | 35 | 94°C, 1 min | 51°C, 1 min | 72°C, 1 min | 72°C, 10 min |
| E | g20-F | g20-R | 1.25 | 250 | 1.5 | 1.0 | 20.0 | 94°C, 3 min | 35 | 94°C, 15 s | 35°C, 1 min | 73°C, 1 min | 73°C, 10 min |
| F | DNApol-90F | DNApol-355R | 4 | 200 | 0.5 mM for lysates  1.5 mM for extracted DNA | 1.0 | 20.0 | 94°C, 4 min | 35 | 94°C, 1 min | 35°C, 1 min | 72°C, 1 min | 72°C, 10 min |
