# Supplementary material for: Prevalence and Evolution of Core Photosystem II Genes in Marine Cyanobacterial Viruses and Their Hosts
Source: PLoS Biol. 2006 Jul 4;4(8):e234. doi: 10.1371/journal.pbio.0040234 (PMC1484495; doi:10.1371/journal.pbio.0040234)
Supplement: Supplementary file 4 [file pbio.0040234.st004.doc]

**Supplementary Table 4**. PCR primers

| **Short Name** | **Full Name** | **Sequence** | **Source** | **Purpose** |
| --- | --- | --- | --- | --- |
| *psbA*-F | 58-VDIDGIREP-66 | 5′-GTNGAYATHGAYGGNATHMGNGARCC-3′ | Zeidner *et al*. 2003 | *psbA* screening |
| *psbA*-R | 331-MHERNAHNFP-340 | 5′-GGRAARTTRTGNGCRTTNCKYTCRTGCAT-3′ | Zeidner *et al*. 2003 | *psbA* screening |
| Pro-*psbA*-F | Pro-*psbA*-1F | 5’-AACATCATYTCWGGTGCWGT-3’ | Z. Johnson | *psbA* screening |
| Pro-*psbA*-R | Pro-*psbA*-1R | 5’-TCGTGCATTACTTCCATACC-3’ | Z. Johnson | *psbA* screening |
| *psbD*-26F | *psbD*-26Fa | 5'-TTYGTNTTYRTNGGNTGGAGYGG-3' | J. A. Lee and  D. Lindell | *psbD* screening |
|  | *psbD*-26Fb | 5'-TTYGTNTTYRTNGGNTGGTCNGG-3' |  |  |
| *psbD*-54F | *psbD*-54Fa | 5'-GTNACNAGYTGGTAYACNCAYGG-3' | J. A. Lee and  D. Lindell | *psbD* screening |
|  | *psbD*-54Fb | 5'-GTNACNTCNTGGTAYACNCAYGG-3' |  |  |
| *psbD*-308R | *psbD*-308Ra | 5'-YTCYTGNGANACRAARTCRTANGC-3' | J. A. Lee and  D. Lindell | *psbD* screening |
|  | *psbD*-308Rb | 5'-YTCYTGRCTNACRAARTCRTANGC-3' |  |  |
| g20-F | CPS1.1 | 5’-GTAGWATWTTYTAYATTGAYGTWGG-3’ | M.B. Sullivan | *g20* screening |
| g20-R | CPS8.1 | 5’-ARTAYTTDCCDAYRWAWGGWTC-3’ | M.B. Sullivan | *g20* screening |
| DNApol-F | DNApol-90Fa | 5’-GAYACIYTIRYIYTITCIMG-3’ | D. Lindell | *DNApol* screening |
|  | DNApol-90Fb | 5’-GAYACIYTIRYIYTIAGYMG-3’ |  |  |
| DNApol-R | DNApol-355Ra | 5’-GGIAYYTGIGCIARRTTIGG-3’ | D. Lindell | *DNApol* screening |
|  | DNApol-355Rb | 5’-GGIAYRTTIGCIARRTTIGG-3’ |  |  |
