# Supplementary material for: Prevalence and Evolution of Core Photosystem II Genes in Marine Cyanobacterial Viruses and Their Hosts
Source: PLoS Biol. 2006 Jul 4;4(8):e234. doi: 10.1371/journal.pbio.0040234 (PMC1484495; doi:10.1371/journal.pbio.0040234)
Supplement: Supplementary file 5 [file pbio.0040234.st005.doc]

**Supplementary Table 5. Likelihood ratio tests (LRTs) for variable evolutionary rates among branches.**

|  | **H_0_: clock** | |  | **H_1_: no clock** | |  | **LRT** | | |
| --- | --- | --- | --- | --- | --- | --- | --- | --- | --- |
|  | **NP^1^** | ***ℓ*** |  | **NP^1^** | ***ℓ*** |  | **2Δ*ℓ*** | **df** | ***P* value** |
| *psbA* |  |  |  |  |  |  |  |  |  |
| Tree 1 | 63 | -13611.73 |  | 125 | -13370.58 |  | 482.3 | 62 | *P* < 0.0001 |
| Tree 2 | 63 | -13845.70 |  | 125 | -13448.08 |  | 795.2 | 62 | *P* < 0.0001 |
|  |  |  |  |  |  |  |  |  |  |
| *psbD* |  |  |  |  |  |  |  |  |  |
| Tree 1 | 41 | -10045.10 |  | 81 | -9941.05 |  | 208.1 | 40 | *P* < 0.0001 |
| Tree 2 | 41 | -10168.01 |  | 81 | -9956.60 |  | 422.8 | 40 | *P* < 0.0001 |

For both *psbA* and *psbD*, individual sequences exhibiting a signature for intragenic recombination (Suppl. Table 1, 2) were excluded from analysis. Likelihood scores were obtained under stationary HKY85 model combined with a gamma correction for among sites rate variation. All model parameters, including nucleotide frequencies, were estimated by using maximum likelihood. Data analysis included all three codon positions. Models were employed as implemented in the baseml program of the PAML package (Yang, 1997). Tree 1 was obtained by Neighbour-Joining analysis of LogDet distances estimated from all three codon positions. Tree 2 was obtained by Neighbour-Joining analysis of LogDet distances estimated from 1^st^ and 2^nd^ codon positions. For both genes, Tree 1 grouped lineages along lines of similarity in evolutionary rates and compositional biases, and Tree 2 did not.

^1^ NP indicates the number of free branch length parameters [node times in the clock model] in the tree topology.
