# Supplementary material for: Prevalence and Evolution of Core Photosystem II Genes in Marine Cyanobacterial Viruses and Their Hosts
Source: PLoS Biol. 2006 Jul 4;4(8):e234. doi: 10.1371/journal.pbio.0040234 (PMC1484495; doi:10.1371/journal.pbio.0040234)
Supplement: Supplementary file 6 [file pbio.0040234.st006.doc]

**Supplementary Table 6. Likelihood ratio tests (LRTs) for non-stationary frequencies among lineages.**

|  | **H_0_** | |  | **H_1_** | |  | **LRT** | | |
| --- | --- | --- | --- | --- | --- | --- | --- | --- | --- |
|  | **NP^1^** | ***ℓ*** |  | **NP^1^** | ***ℓ*** |  | **2Δ*ℓ*** | **df** | **P value** |
| *psbA*^2^ |  |  |  |  |  |  |  |  |  |
| Tree 1 | 3(1) | -10459.77 |  | 15(5) | -10274.38 |  | 370.8 | 12 | *P* < 0.0001 |
| Tree 2 | 3(1) | -10512.53 |  | 15(5) | -10332.88 |  | 359.3 | 12 | *P* < 0.0001 |
|  |  |  |  |  |  |  |  |  |  |
| *psbD*^3^ |  |  |  |  |  |  |  |  |  |
| Tree 1 | 3(1) | -9941.05 |  | 18(6) | -9785.28 |  | 311.5 | 15 | *P* < 0.0001 |
| Tree 2 | 3(1) | -9956.60 |  | 18(6) | -9808.98 |  | 295.2 | 15 | *P* < 0.0001 |

H_0_ denotes the null hypothesis of stationary nucleotide frequencies; this was modelled by specifying one set of nucleotide frequencies for all branches of the tree. H_1_ denotes the alternative hypothesis of non-stationary nucleotide frequencies; this was modelled by assigning all branches of the tree topology to one of several independent sets of frequency parameters (6 sets for *psbA* and 5 sets for *psbD*). Apart from nucleotide frequencies, H_0_ and H_1_ assumed a substitution process equivalent to an HKY85 model combined with a gamma model for among sites rate variation. The transition to transversion ratio was assumed to be homogenous among branches. H_1_ represents a user-defined version of the non-homogenous models of Yang and Roberts (1995). All model parameters, including nucleotide frequencies, were estimated by using maximum likelihood. Data analysis included all three codon positions. Models were employed as implemented in the baseml program of the PAML package (Yang, 1997).

Tree 1 was obtained by Neighbour-Joining analysis of LogDet distances estimated from all three codon positions. Tree 2 was obtained by Neighbour-Joining analysis of LogDet distances estimated from 1^st^ and 2^nd^ codon positions. For both genes, Tree 1 grouped lineages along lines of similarity in evolutionary rates and compositional biases, and Tree 2 did not. User defined sets of frequency parameters for H_1_ were specified in the tree file (shown below) by using the “branch label” format described in the PAML manual. For both *psbA* and *psbD*, individual sequences exhibiting a signature for intragenic recombination (Suppl. Tables 1, 2) were excluded from analysis.

^1^ NP indicates the number of free parameters for nucleotide frequencies. Number in parentheses indicates the number of independent sets (A+C+G+T) of user specified frequency parameters.

^2^*psbA* Tree 1:

((((Syncy_3,Syncy_2),(Anab_1,(Anab_3,(Anab_4,Anab_2)))),(Gloe_A,(Thermo_2,(Thermo_3,Thermo_1)))),(((WH8101_B #1,(((WH8103_A #1,(WH8102_B #1,WH8102_D #1)#1)#1,((WH8012 #1,((Syn9902_psbA1_Syn9902_psbA2_Syn9902_psbA3 #1,Syn9902_psbA4 #1)#1,(WH8020_C #1,WH8020_B #1)#1)#1)#1,(WH8109 #1,(Syn9605_psbA2_Syn9605_psbA3 #1,SynRSS9907 #1)#1)#1)#1)#1,(SynRS9901_1 #1,(WH7803 #1,(WH8018_C #1,(WH8018_B #1,(WH8017 #1,WH8018_A #1)#1)#1)#1)#1)#1)#1),(MIT9313 #1,MIT9303 #1)#1)#1,((syn1_8101 #2,(PSSM1_9303 #2,((SBM4 #2,(syn19_8109 #2,(SSSM5_SSSM3_8018 #2,syn30_8018 #2)#2)#2)#2,((SRSM28_7803 #2,SWHM1_7803 #2)#2,(syn10_8017_Syn26_8017 #2,syn9_8109 #2)#2)#2)#2)#2)#2,((BAC9D04 #2,(SPM2_7803 #2,SRSM88_7803 #2)#2)#2,(((PGSP1_MED4 #3,(PSSP5_9515 #3,PSSP7_MED4 #3)#3)#3,(PSSP3_9312 #3,PSSP6_9515 #3)#3)#3,((NATL2A #3,(MIT9211 #3,SS120 #3)#3)#3,((PSSM8_9211 #3,(PRSM5_1A #3,(PSSM2_NATL1A #3,PSSM3_2A #3)#3)#3)#3,(((MED4 #3,MIT9515 #3)#3,(MIT9302 #3,MIT9312 #3)#3)#3,((PSSM4_2A_PRSM2_2A #3,(SShM1_6501 #3,SSSM1_6501 #3)#3)#3,(PSSM10_2A #3,PRSM4_9303 #3)#3)#3)#3)#3)#3)#3)#2)#2)#4);

^2^*psbA* Tree 2:

((((Syncy_3,Syncy_2),(Anab_1,(Anab_3,(Anab_4,Anab_2)))),(Gloe_A,(Thermo_2,(Thermo_3,Thermo_1)))),((WH8101_B #1,((((syn1_8101 #2,(PSSM1_9303 #2,(((syn19_8109 #2,(SSSM5_SSSM3_8018 #2,syn30_8018 #2)#2)#2,(SBM4 #2,(SRSM28_7803 #2,SWHM1_7803 #2)#2)#2)#2,(syn10_8017_Syn26_8017 #2,syn9_8109 #2)#2))#2)#2,(BAC9D04 #2,(SPM2_7803 #2,SRSM88_7803 #2)#2)#2)#2,((WH8102_D #1,(WH8102_B #1,WH8103_A #1)#1)#1,((Syn9902_psbA1_Syn9902_psbA2_Syn9902_psbA3 #1,(WH8020_C #1,(Syn9902_psbA4 #1,(WH8020_B #1,WH8012 #1)#1)#1)#1)#1,(SynRSS9907 #1,(Syn9605_psbA2_Syn9605_psbA3 #1,WH8109 #1)#1)#1)#1)#1)#1,(SynRS9901_1 #1,(WH8018_B #1,(WH7803 #1,(WH8018_A #1,(WH8017 #1,WH8018_C #1)#1)#1)#1)#1)#1)#1)#1,(((PGSP1_MED4 #3,(PSSP5_9515 #3,PSSP7_MED4 #3)#3)#3,(PSSP3_9312 #3,PSSP6_9515 #3)#3)#3,((MIT9313 #3,MIT9303 #3)#3,((MIT9211 #3,SS120 #3)#3,((NATL2A #3,((PSSM2_NATL1A #3,PSSM3_2A #3)#3,(PRSM5_1A #3,PSSM8_9211 #3)#3)#3)#3,(((MED4 #3,MIT9515 #3)#3,(MIT9302 #3,MIT9312 #3)#3)#3,(PSSM10_2A #3,(PRSM4_9303 #3,(PSSM4_2A_PRSM2_2A #3,(SShM1_6501 #3,SSSM1_6501 #3)#3)#3)#3)#3)#3)#3)#3)#3)#3)#4);

^3^*psbD* Tree 1:

((Gloe,(((Syncy,Syncy_2),(Arabidopsis,(Anab_A,Anab_B))),(Thermo_2,Thermo_1))),((((WH8101 #1,((Syn9605_1_Syn9605_2 #1,WH8109 #1)#1,(WH8012 #1,(WH8102_1_WH8102_2 #1,WH8103 #1)#1)#1)#1)#1,(Syn9902_1_Syn9902_2 #1,WH8020 #1)#1)#1,(MIT9313 #1,MIT9303 #1)#1)#1,((((SSSM2 #2,SShM2_8102 #2)#2,(syn1_8101 #2,Syn33_7803 #2)#2)#2,(SPM2_7803 #2,((SRSM2_7803 #2,(SBM4 #2,(syn9_8012 #2,(syn10_8017 #2,Syn26_8017 #2)#2)#2)#2)#2,(SSSM5_8102 #2,((SSM1_6501 #2,syn19_8109 #2)#2,(SSSM3_8018 #2,syn30_8018 #2)#2)#2)#2)#2)#2)#2,((MED4 #3,(MIT9515 #3,(MIT9302 #3,(MIT9215 #3,MIT9312 #3)#3)#3)#3)#3,((NATL2A #3,NATL1A #3)#3,(SS120 #3,MIT9211 #3)#3)#3)#3)#4)#5);

^3^*psbD* Tree 2:

((Gloe,((Syncy,Syncy_2),((Arabidopsis,(Anab_A,Anab_B)),(Thermo_2,Thermo_1)))),(((SPM2_7803 #2,(SBM4 #2,(((Syn33_7803 #2,(syn1_8101 #2,(SSSM2 #2,SShM2_8102 #2)#2)#2)#2,(SSSM3_8018 #2,syn30_8018 #2)#2)#2,((SRSM2_7803 #2,(syn9_8012 #2,(syn10_8017 #2,Syn26_8017 #2)#2)#2)#2,(SSSM5_8102 #2,(SSM1_6501 #2,syn19_8109 #2)#2)#2)#2)#2)#2)#2,(WH8020 #1,(WH8101 #1,((Syn9605_1_Syn9605_2 #1,(WH8109 #1,Syn9902_1_Syn9902_2 #1)#1)#1,(WH8012 #1,(WH8102_1_WH8102_2 #1,WH8103 #1)#1)#1)#1)#1)#1)#4,((MIT9313 #3,MIT9303 #3)#3,((MED4 #3,(MIT9515 #3,(MIT9302 #3,(MIT9215 #3,MIT9312 #3)#3)#3)#3)#3,((NATL2A #3,NATL1A #3)#3,(SS120 #3,MIT9211 #3)#3)#3)#3)#3)#5);
