# Supplementary material for: Prevalence and Evolution of Core Photosystem II Genes in Marine Cyanobacterial Viruses and Their Hosts
Source: PLoS Biol. 2006 Jul 4;4(8):e234. doi: 10.1371/journal.pbio.0040234 (PMC1484495; doi:10.1371/journal.pbio.0040234)
Supplement: Supplementary file 7 [file pbio.0040234.st007.doc]

**Supplementary Table 7**. Likelihood-based statistical comparison of competing evolutionary hypotheses under a model of non-stationary nucleotide frequencies.

| **Data partition** | ***ℓ*** | **Δ*ℓ*** | ***P*_KH_** | ***P*_SH_** | ***P*_RELL_** |
| --- | --- | --- | --- | --- | --- |
| *psbA*, All sites |  |  |  |  |  |
| Tree 1 | -13147.50 | NA | NA | NA | 0.75 |
| Tree 2 | -13182.14 | -34.6 | 0.245 | 0.252 | 0.25 |
|  |  |  |  |  |  |
| *psbA*, 1^st^ and 2^nd^ codon positions |  |  |  |  |  |
| Tree 1 | -4117.33 | -90.4 | 0.004 | 0.004 | 0.003 |
| Tree 2 | -4026.88 | NA | NA | NA | 0.997 |
|  |  |  |  |  |  |
| *psbD*, All sites |  |  |  |  |  |
| Tree 1 | -9785.28 | NA | NA | NA | 0.76 |
| Tree 2 | -9808.98 | -23.7 | 0.239 | 0.239 | 0.24 |
|  |  |  |  |  |  |
| *psbA*, 1^st^ and 2^nd^ codon positions |  |  |  |  |  |
| Tree 1 | -3020.89 | -45.770 | 0.012 | 0.016 | 0.009 |
| Tree 2 | -2975.12 | NA | NA | NA | 0.991 |

*P*_KH_ denotes the *P*-value for the KH normal test of Kishino and Hasegawa (1989). *P*_SH_ denotes the *P*-value for the SH test (Shimodaira and Hasegawa, 1999). *P*_RELL_ denotes the RELL bootstrap proportion (Kishino and Hasegawa, 1989). Note that although tree 1 and tree 2 were not selected independently of the data, neither was selected according to its likelihood score. For both genes, Tree 1grouped lineages along lines of similarity in evolutionary rates and compositional biases, and Tree 2 did not. For both *psbA* and *psbD*, individual sequences exhibiting a signature for intragenic recombination (Suppl. Tables 1, 2) were excluded from analysis. Tree 1 was estimated by a Neighbour-Joining analysis of LogDet distances from all sites, and Tree 2 was estimated by a Neighbour-Joining analysis of LogDet distances based on only 1^st^ and 2^nd^ codon positions. Likelihood scores were obtained under non-stationary models of nucleotide frequencies (see Suppl. Table 5 for additional model details).
